# Correction: Rashid et al. Natural Compounds of *Lasia spinosa* (L.) Stem Potentiate Antidiabetic Actions by Regulating Diabetes and Diabetes-Related Biochemical and Cellular Indexes. *Pharmaceuticals* 2022, *15*, 1466

**DOI:** 10.3390/ph16020256

**Published:** 2023-02-08

**Authors:** Md. Mamunur Rashid, Md. Atiar Rahman, Md. Shahidul Islam, Md. Amjad Hossen, A. M. Abu Ahmed, Mirola Afroze, Alaa H. Habib, Manal M. S. Mansoury, Hend F. Alharbi, Reham M. Algheshairy, Walla Alelwani, Afnan M. Alnajeebi, Jitbanjong Tangpong, Srabonti Saha, Alaa Qadhi, Wedad Azhar

**Affiliations:** 1 Department of Biochemistry and Molecular Biology, University of Chittagong, Chittagong 4331, Bangladesh; 2 School of Allied Health Sciences, Walailak University, Nakhon Si Thammarat 80160, Thailand; 3 Department of Pharmacy, Faculty of Science and Engineering, International Islamic University Chittagong, Chittagong 4318, Bangladesh; 4 Department of Genetic Engineering and Biotechnology, University of Chittagong, Chittagong 4331, Bangladesh; 5 Bangladesh Reference Institute for Chemical Measurements (BRiCM), Dr. Qudrat-e-Khuda Road (Laboratory Road), Dhanmondi, Dhaka 1205, Bangladesh; 6 Department of Physiology, Faculty of Medicine, King Abdulaziz University, Jeddah 21589, Saudi Arabia; 7 Department of Food and Nutrition, Faculty of Human Sciences and Design, King Abdulaziz University, Jeddah 21589, Saudi Arabia; 8 Department of Food Science and Human Nutrition, College of Agriculture and Veterinary Medicine, Qassim University, Buraydah 51452, Saudi Arabia; 9 Department of Biochemistry, Collage of Science, University of Jeddah, Jeddah 80203, Saudi Arabia; 10 Clinical Nutrition Department, Faculty of Applied Medical Sciences, Umm Al-Qura University, P.O. Box 715, Makkah 21955, Saudi Arabia

In the published publication [1], there was an error regarding the affiliation for Manal M. S. Mansoury. The correct affiliation should be the newly added affiliation 7:

^7^ Department of Food and Nutrition, Faculty of Human Sciences and Design, King Abdulaziz University, Jeddah 21589, Saudi Arabia

The authors state that the scientific conclusions are unaffected. This correction was approved by the Academic Editor. The original publication has also been updated.
