# Supplementary material for: Natural Compounds of Lasia spinosa (L.) Stem Potentiate Antidiabetic Actions by Regulating Diabetes and Diabetes-Related Biochemical and Cellular Indexes
Source: Pharmaceuticals (Basel). 2022 Nov 25;15(12):1466. doi: 10.3390/ph15121466 (PMC9781412; doi:10.3390/ph15121466)
Supplement: Supplementary file 1 [file pharmaceuticals-15-01466-s001.zip › pharmaceuticals-2054104-supplementary.pdf]

Supplementary Table S1. Phytochemical group tests of MExLS.

Phytochemical Constituents	Test Name	Observations of MExLS Extract
**Carbohydrates**	Molisch's test	+
	Benedict test	+
**Alkaloids**	Mayer's test	+
	Wagner's test	+
	Dragendorff's test	+
**Cardiac glycosides**	Molisch's test	+
	Keller-Killani test	–
**Carbohydrates**	Benedict test	+
**Flavonoids**	Alkali test	+
	Lead (II) acetate	+
**Steroids**	Salkowski's test	+
	Liberman test	+
**Saponin**	Frothing test	–
**Tannins**	General Color test	+
**Phlobatannins**	General color test	–
**Protein**	Biruet's test	+
	Ninhydrin Test	+

The phytochemical status of the crude MExLS has been investigated and the results are summarized in the supplementary Table S1. Phytochemical groups indicate the nature of phytoconstituents whether present or absent in the crude extract of methanol extract of *Lasia*

spinosa. Tests were accomplished using established methods. The presence and absence of the phytochemicals are denoted using the signs (+) and (–), respectively.
